# The Best DFT Functional Is the Ensemble of Functionals

**DOI:** 10.1002/advs.202408239

**Published:** 2024-10-25

**Authors:** Yuting Rui, Yuxinxin Chen, Elena Ivanova, Vignesh Balaji Kumar, Szymon Śmiga, Ireneusz Grabowski, Pavlo O. Dral

**Affiliations:** ^1^ State Key Laboratory of Physical Chemistry of Solid Surfaces College of Chemistry and Chemical Engineering Fujian Provincial Key Laboratory of Theoretical and Computational Chemistry Xiamen University Xiamen Fujian 361005 China; ^2^ Chair of Statistics School of Business and Economics Humboldt University of Berlin Unter den Linden 6 10099 Berlin Germany; ^3^ Institute of Physics Faculty of Physics, Astronomy, and Informatics Nicolaus Copernicus University in Toruń ul. Grudziądzka 5 Toruń 87‐100 Poland

**Keywords:** ensemble learning, density functional theory, DFT calculations

## Abstract

The development of better density functional theory (DFT) methods is one of the most active research areas, given the importance of DFT for ubiquitous molecular and materials simulations. However, this research primarily focuses on improving a specific exchange‐correlation Kohn–Sham density functional. Here, a robust procedure is proposed for constructing transferable ensembles of density functionals that perform superior to any constituent individual density functional. It is shown that such ensembles built only with the density functionals predating the GMTKN55 benchmark of 2017 can reach a record‐low weighted error of 1.62 kcal mol^−1^ on this benchmark compared to 3.08 kcal mol^−1^ of the best constituent density functional. The DENS24 density functional ensembles are also introduced as practical DFT methods with consistently accurate performance for various simulations at affordable cost. DENS24 ensembles are open‐source and can be used for simulations online. Additionally, it is shown that the ensembles can be integrated into the SCF procedure by creating mixed DENS24 functionals, which have the same accuracy but are faster than ensembles of independent functionals.

## Graphical abstract

DENS24 is the density functional ensembles combining predictions from multiple density functional theory (DFT) functionals to yield higher accuracy than individual functionals have. The more functionals are in ensemble—the more accurate your calculations.

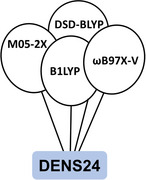

## 1 Introduction

Density functional theory (DFT)^[^ ^1^, ^2^ ^]^ is the most common quantum mechanical framework widely used in molecular and materials simulations. DFT methods are typically based on approximate forms of density functionals exhibiting different applicability and accuracy in various systems and property calculations. Selecting an appropriate exchange‐correlation Kohn–Sham (KS) functional^[^ ^3^, ^4^ ^]^ is crucial for obtaining accurate computational results, making developing and applying DFT as one of the research hotspots in theoretical chemistry. Despite the increasing variety of functionals and improving computational accuracy, defining the “best” functional remains challenging. Ideally, a functional should be computationally efficient, highly accurate, universal, and versatile enough to apply to various systems and problems. However, to date, such an optimal functional has not been developed.

Here, we propose to leverage the power of ensemble learning^[^ ^5^, ^6^ ^]^ to optimally combine existing density functionals rather than design an optimal individual functional. Ensemble learning combines predictions from multiple models using machine learning techniques to generate a more robust and accurate predictive model, effectively harnessing the strengths of various models while mitigating the weaknesses of individual ones and thus enhancing the overall prediction accuracy and robustness. Here, we focus on applying ensemble learning to combine the final properties (exemplified by energies) calculated independently by individual density functionals. This way, our implementations can be easily adapted to any set of functionals without the need to modify them. That is in contrast to an ensemble DFT focusing on ensembles of different electronic states, so specialized algorithms and implementations are required to alter the DFT method.^[^ ^7^ ^]^ We will also show that our approach can be used to obtain weights for constructing the general‐purpose, mixed exchange‐correlation functionals inspired by the earlier works mixing up to two exchange‐correlation functionals for specific applications.^[^ ^8^ ^]^

In this work, we introduce the procedure for constructing a robust density functional ensembles (DENS) and demonstrate its application for total ground‐state energies of diverse compounds. The DENS procedure delivers methods that are more accurate in the target properties by construction than any individual density functional entering the ensemble. Beyond the benchmarks, we also develop the series of DENS24 ensembles which, can be used as a robust replacement for individual DFT methods in typical simulations, from energy calculations to geometry optimizations, vibrational spectra and thermochemistry, and molecular dynamics.

## 2 Methods

### 2.1 Density Functional Ensemble

Here, we suggest treating each density functional as a “weak learner” and combining their predictions into an ensemble to obtain a “strong learner.”^[^ ^6^ ^]^ Each functional has advantages and disadvantages, and the errors are often random. Hence, by combining many functionals, the amount of random error can be reduced, which is ensured by the known laws of statistics.^[^ ^9^, ^10^ ^]^

Focusing on calculating total energy *E*, the key idea behind density functional ensembles is that we want to find the best function *f* taking as input the total energies *E_i_* calculated with the *N* density functionals entering the ensemble:

(1) $$E=f\left({\left\{E_{i}\right\}}_{i=1}^{N}\right)$$

Many possible choices for *f* are possible, including two extremes—simple averaging and highly flexible neural networks, both of which were initially explored. Simple averaging works to some extent, but it is obviously not the best solution, as some functionals are better than others, and many errors of functionals are systematic. On the other hand, solutions such as neural networks are nonlinear and too flexible. At the same time, the ensemble must satisfy the essential conditions of robustness (transferability) even when the ensemble is trained on limited data. In addition, the ensemble should be size‐consistent^[^ ^11^ ^]^ to the extent individual density functionals are (individual density functionals might not be size‐consistent, but that's beside the point because the ensemble approach should not make it worse by design). Neural networks would not satisfy this condition. Another practical consideration is that the ensemble should be suitable for straightforward applications to simulations such as geometry optimization, frequency calculations, and dynamics, all of which require energy derivatives (energy gradients = negative forces, Hessians, etc.).

Following the principle of Occam's razor, the simplest function is adopted that satisfies the needs of the linear regression, which can be viewed as a weighted average:

(2) $$E=\sum_{i=1}^{N}\omega_{i}E_{i}$$

where ω_*i*_ are functional‐specific weights.

Simple derivations show that this equation perfectly satisfies the requirement for a simple solution to obtain energy derivatives, e.g., for forces:

(3) $$F=-\;\frac{\partial E}{\partial R}=-\sum_{i=1}^{N}\omega_{i}\frac{\partial E_{i}}{\partial R}$$

and size‐consistency, e.g., for a system consisting of two subsystems, *A* and *B*, at large separation:

(4) $$E=E^{A}+E^{B}=\sum_{i=1}^{N}\omega_{i}E_{i}^{A}+\sum_{i=1}^{N}\omega_{i}E_{i}^{B}$$

if

(5) $$E_{i}=E_{i}^{A}+E_{i}^{B}$$

The ω_*i*_ coefficients can be obtained with standard statistical methods. However, we need to ensure proper regularization, preventing too large weights from leading to numerical instabilities and potentially overfitting. We tested Lasso,^[^ ^12^ ^]^ ridge,^[^ ^13^ ^]^ and LarsLasso^[^ ^14^ ^]^ regressions for regularization (as implemented in scikit‐learn^[^ ^15^ ^]^) and searched for the regularization parameters based on the 8:2 splitting of the small subset of the training data into the training and validation sets. In the end, for all the results in this work, we used the ridge regression with the hyperparameter *ɑ* = 10. A significant additional advantage of the regularization methods is that the importance of each functional can be judged based on its weight in the ensemble (reported in the Supporting Information, Excel file, and in the open‐source implementation of the ensembles in MLatom,^[^ ^16^ ^]^ version ≥3.11, https://github.com/dralgroup/mlatom).

The weights obtained with the regression methods do not necessarily add up to the unity, although the tests indicate that the sum of weights is almost unity. This is not a problem for calculating energies and derivatives of energies, but when evaluating properties such as electron density or building mixed exchange‐correlation functionals, we need to ensure that the sum of the weights is equal to one. For this, the weights ω_*i*_ obtained via regression are normalized as:

(6) $${\hat{\omega }}_{i}=\omega_{i}\;/\sum_{j=1}^{N}\omega_{i}$$

### 2.2 Training Ensembles

Ensembles must be trained, i.e., we need to find as many parameters as we have functionals in an ensemble. For training, the sets are used from the standard GMTKN55 database,^[^ ^17^ ^]^ which encompasses 1505 reference energies for reactions and barrier heights in main‐group and organic chemistry. These values are derived from high‐level wavefunction calculations sourced from over fifty references. The database comprises 55 subsets categorized into five groups representing various chemical reaction types: fundamental properties, reactions, and isomerizations involving larger systems, barrier heights, and inter‐ and intra‐molecular noncovalent interactions.

The figure of merit in the GMTKN55 benchmark is the so‐called weighted total mean absolute deviation‐2 (WTMAD‐2), taking into account the different scales of different types of reaction energies:

(7) $$\mathrm{WTMAD}-2\;=\frac{1}{\sum_{i}^{55}N_{i}}\;\sum_{i}^{55}N_{i}\frac{56.84\;\mathrm{kcal}\;{\mathrm{mol}}^{-1}}{{\bar{\left|\Delta E\right|}}_{i}}\cdot \mathrm{MA}D_{i}$$

where the weighting factor is based on the ratio between the average of all 55 |Δ*Ε*| values (56.84 kcal mol^−1^) and the |Δ*Ε*| values of each respective test set. Then, this ratio is scaled by the relative number of energy points *N_i_* in a specific set. The weighted average absolute deviation for all data points is summed, and the result is divided by the total number of relative energies in GMTKN55 (1505 data points) to obtain the WTMAD‐2 (Equation 7) for the entire dataset. This expression can also be adapted to different compositions of subsets.

While training, there is also need to account for the different scales in relative energies in different subsets and, hence, the energies are scaled in each subset by the same weighting factor of $56.84\;\mathrm{kcal}\;{\mathrm{mol}}^{-1}/{\bar{\left|\Delta E\right|}}_{i}$, similarly as in the WTMAD‐2. Note, however, that the actual loss function is the weighted sum of residual squared errors, i.e., our training procedure does not guarantee the lowest WTMAD‐2. Using the lowest weighted sum of residual squared errors is arguably better to minimize outliers.

## 3 Results

### 3.1 Making the Statistically Best Functional

We start with a proof‐of‐concept demonstration of the power of our density functional ensemble (DENS) procedure by achieving the statistically best result for the standard GMTKN55 benchmark^[^ ^17^ ^]^ of the DFT methods. At the time of the introduction of the GMTKN55 benchmark, it was used to evaluate 84 functionals. Since then, it has been used to assess or even design better functionals. Inevitably, those later functionals reported better performance on the GMTKN55 benchmark. Hence, for a fair comparison, we decided to use only the functionals chosen among the 84 originally evaluated ones for building the ensembles.

**Figure 1:**
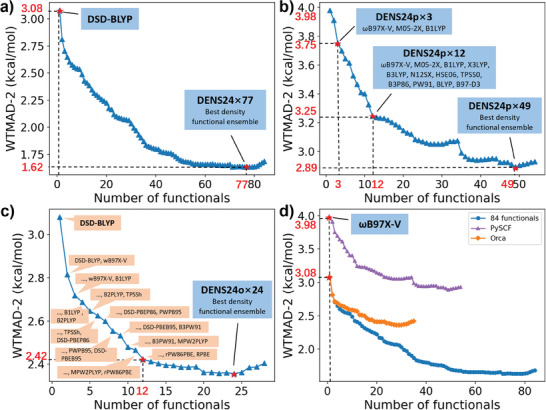
Performance of different versions of the density functional ensembles on GMTKN55. The number *N* in DENS24×*N* denotes the number of functionals used.

We construct the DENS24 ensembles with the forward stepwise selection scheme by starting with the DSD‐BLYP‐D3(BJ)^[^ ^18^ ^]^ functional, which had the smallest WTMAD‐2 in the original benchmark.^[^ ^17^ ^]^ At each iteration, a new functional is added, which brings the biggest reduction in WTMAD‐2. The forward stepwise selection finds the best combination of 2–4 functionals, as we validated by brute‐force evaluation on all possible combinations of this small number of functionals. This procedure delivers the series of the DENS24×*N* density functional ensembles comprising the increasing number *N* of individual functionals. The WTMAD‐2 generally drops with more functionals (**Figure** **1**a), but after 77 functionals it slightly goes up (because our fitting targets the weighted sum of the residual squared errors rather than WTMAD‐2). The number of functionals *N* in DENS24×*N* can be considered as a parameter to choose, similarly to the choice of the basis set, i.e., generally, the larger the better.

The DENS24×77 density functional ensemble reaches only 1.62 kcal mol^−1^ error—quite close to the coveted chemical accuracy (error of 1 kcal mol^−1^). It is quite impressive given that the training ensemble optimizes only 77 weights on 1505 points, minimizing the possible effects of overfitting. To put this value into perspective, the best individual functional in the ensemble (DSD‐BLYP‐D3(BJ)^[^ ^18^ ^]^) has an almost twice larger error of 3.08 kcal mol^−1^ (Figure 1a). Some of the newer functionals, which were introduced after the original GMTKN55 publication, have either close or much higher errors, e.g., very recent DH23 and revDH23^[^ ^19^ ^]^ have WTMAD‐2 of 1.73–1.76 kcal mol^−1^, DM21^[^ ^20^ ^]^ and CF22D^[^ ^21^ ^]^ methods (which used much more complex neural networks for improvement) have WTMAD‐2 of 3.97 and 3.64, respectively. By construction, our ensemble approach can reach even smaller WTMAD‐2 values if we include those newest functionals.

### 3.2 Transferability of Ensembles

One of the major concerns is how transferable the ensembles are. Hence, we explore whether good ensembles can be built with a limited number of functionals and trained on a few subsets. We have manually chosen 10 out of 55 subsets (18%), which represent as diverse as possible selection of reactions: MB16‐43,^[^ ^22^ ^]^ G21EA,^[^ ^16^, ^23^ ^]^ G21IP,^[^ ^16^, ^23^ ^]^ ISOL24,^[^ ^24^ ^]^ G2RC,^[^ ^16^, ^25^ ^]^ ISO34,^[^ ^26^ ^]^ W4‐11,^[^ ^16^, ^23^, ^27^ ^]^ S66,^[^ ^28^ ^]^ IDISP,^[^ ^16^, ^23^ ^]^ and DARC.^[^ ^16^, ^29^ ^]^ The selection was based on human evaluation and, independently, MB16‐43 was recently recommended^[^ ^30^ ^]^ as a critical ingredient to increase transferability when fitting individual density functionals. For the choice of functionals, we screened out 54 hybrid functionals supported by the open‐source PySCF^[^ ^16^, ^25^ ^]^ from a pool of 84 functionals. After we fitted the DENS with 54 functionals on ten subsets, we identified the top eight functionals by their weight in the ensemble: B3LYPG,^[^ ^31^ ^]^ MN12SX,^[^ ^32^ ^]^ PBE0,^[^ ^33^ ^]^ PW91,^[^ ^34^, ^35^ ^]^ revPBE,^[^ ^36^ ^]^ MN15,^[^ ^37^ ^]^ SOGGA11X,^[^ ^38^ ^]^ and MN12L.^[^ ^32^ ^]^

**Figure 2:**
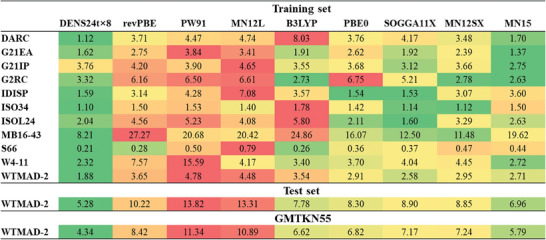
Performance of the eight functionals and their DENS24t×8 ensemble for the training, test, and entire GMTKN55 sets.

These eight functionals were used to form a new DENS24t×8 method that has the smallest WTMAD‐2 of 1.88 kcal/mol for the ten subsets used for fitting compared to the 2.58–4.78 kcal mol^−1^ of the individual functionals (**Figure** **2**). The most critical test comes by evaluating the DENS24t×8 functional's performance on the entire GMTKN55: the DENS24t×8 WTMAD‐2 is only 4.34 kcal mol^−1^ compared to 5.79–11.34 kcal mol^−1^ errors of the individual functionals. Also, the error on the 45 test subsets not used in the training, is the lowest for the ensemble compared to the constituting functionals. These tests validate the transferability of our approach.

### 3.3 DENS24 as a Practical Realization of Density Functional Ensembles

After we demonstrated the transferability of our approach by limiting ourselves to 18% of the entire GMTKN55 data set, we can apply it with confidence for the practical implementation of the ensembles for general‐purpose applications in chemical simulations. We construct two series of ensembles using the above forward stepwise selection scheme. The first series denoted DENS24o×*N* is based on the functionals supported by a popular Orca^[^ ^39^ ^]^ program and the second series denoted DENS24p×*N*—on functionals provided by the open‐source PySCF (excluding double‐hybrid ones). Both series are implemented in the open‐source MLatom^[^ ^40^ ^]^ package (Python code available at https://github.com/dralgroup/mlatom), and the calculations with the open‐source PySCF‐based series can be performed on the XACS online computing platform.^[^ ^41^ ^]^

**Figure 3:**
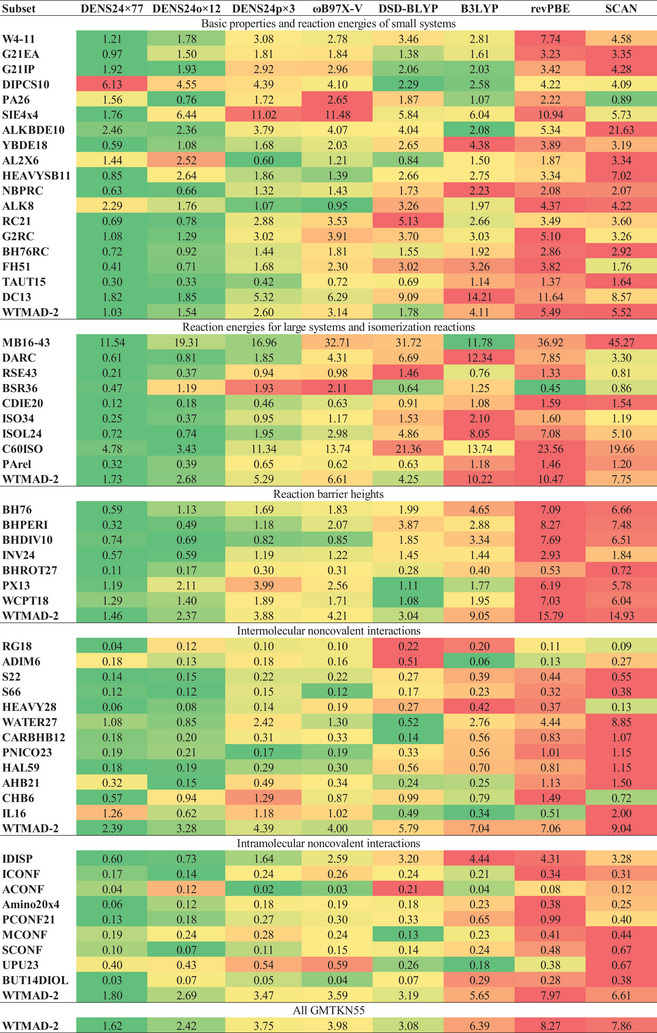
The performance of the various density functional ensembles constructed in this work compared to selected individual functionals on the GMTKN55 sets.

The Orca‐based series achieves the minimum WTMAD‐2 of 2.36 kcal mol^−1^ with 24 functionals (Figure 1c). However, practically speaking, with 12 functionals we achieve almost the same result with WTMAD‐2 of 2.42 kcal mol^−1^ and, therefore, we recommend it as the maximum number of functionals to be used with this series (**Figure** **3**). Interestingly, already the smallest DENS24o×2 ensemble consists of the best double‐hybrid (DSD‐BLYP‐D3(BJ)) and the best hybrid (ωB97X‐V^[^ ^42^ ^]^) functionals and substantially reduces the error to ca. 2.8 kcal mol^−1^.

The PySCF‐based series does not use any double‐hybrid functionals and, hence, has generally higher errors (Figure 1b,d). The lowest error in this series is 2.89 kcal mol^−1^, achieved with 49 functionals. DENS24p×3 (ωB97X‐V,^[^ ^42^ ^]^ B1LYP,^[^ ^43^ ^]^ M05‐2X^[^ ^44^ ^]^) may serve as a cheap option giving the largest error drop—its WTMAD‐2 is 3.75 compared to 3.98 kcal mol^−1^ of ωB97X‐V (Figure 3). DENS24p×12 achieves WTMAD‐2 of 3.25 kcal mol^−1^, but it takes 21 hybrid functionals to achieve the WTMAD‐2 of 3.09 kcal mol^−1^ close to the best double‐hybrid functional DSD‐BLYP‐D3(BJ).

Overall, compared with each component functional, the ensembles have fewer outliers and narrower error distributions. Detailed information on the performance of the whole GMTKN55 for several versions of DENS24p×12 is shown in Notes S1–S3 (Supporting Information).

### 3.4 Effect of the Basis Set

**Figure 4:**
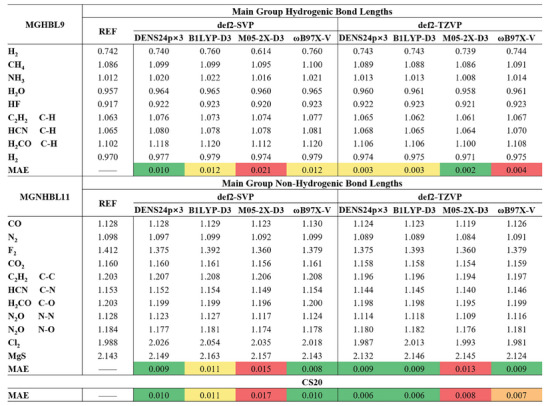
Geometry optimization with DENS24p×3 and its component functionals. The unit of bond length is Ångstrom.

Since the quadruple‐𝜁 basis set is not the common choice for most types of calculations, we applied the weights fitted on the quadruple‐𝜁 basis set to the triple‐ and double‐𝜁 basis sets. We choose Ahlrichs’ split‐valence Gaussian atomic‐orbital basis sets def2‐SVP and def2‐TZVP^[^ ^45^ ^]^ and test DENS24p×3/def2‐SVP and DENS24p×3/def2‐TZVP on geometry optimization of MGHBL9 and MGNHBL11,^[^ ^46^ ^]^ which contain a series of simple compounds with reference bond lengths of hydrogenic and non‐hydrogenic bond. Explicit D3(BJ)^[^ ^47^, ^48^ ^]^ dispersion correction is used as recommended in previous work^[^ ^17^ ^]^ for functionals without embedded dispersion corrections. Benchmark results (**Figure** **4**) show that the mean absolute error of bond lengths predicted by DENS24p×3/def2‐SVP is around 0.01 Å, and with def2‐TZVP even lower.

Beyond geometry optimization, we qualitatively checked whether DENS24 can give a reasonable description of the potential energy curve of the C─H bond in methane (before dissociation where single‐reference DFT methods struggle^[^ ^49^, ^50^ ^]^) (Note S4, Supporting Information). The dissociation curve possesses good physical agreement with the location of the minima and no energy fluctuation or jumps are observed, indicating the advantages of using linear regression in ensembles instead of more complex fitting tools, such as neural networks, which may require additional constraints to ensure physical correctness.^[^ ^51^ ^]^

## 4 Discussion

### 4.1 Electron Density of Ensembles

In addition to evaluating the functional performance on reaction energies, it is crucial to analyze the adequacy of electron densities to judge the quality of a functional.^[^ ^52^ ^]^ We define the electron density of the ensembles as the weighted sum of densities obtained with individual functionals, e.g.:

(8) $$\rho_{\mathrm{DENS}}\;\left(r\right)=\sum_{i=1}^{N}{\hat{\omega }}_{i}\rho_{i}\left(r\right)$$

where ${\hat{\omega }}_{i}$ is the normalized weight for the corresponding functional.

We evaluate the DENS24p×*N* series on the CCSD(T) relaxed densities of 16 systems are including 11 molecules and 5 atoms (Ar, Be, C_2_H_6_, Cl_2_, CO, H_2_, H_2_O, HCl, He, He_2_, HF, Mg, N_2_, Ne, Ne_2_ and NH_3_).^[^ ^53^ ^]^ All of these systems are presented in GMTKN55 but the target of fitting in DENS functionals were energies and not densities. The reference values of electron density were generated with uncontracted aug‐cc‐pVTZ. The relative errors of electron densities compared with reference is quantified as:

(9) $$\Delta \rho =\frac{∥\rho_{\mathrm{ref}}-\rho_{\mathrm{DENS}}{∥}_{L2}}{∥\rho_{\mathrm{ref}}{∥}_{L2}}$$

**Figure 5:**
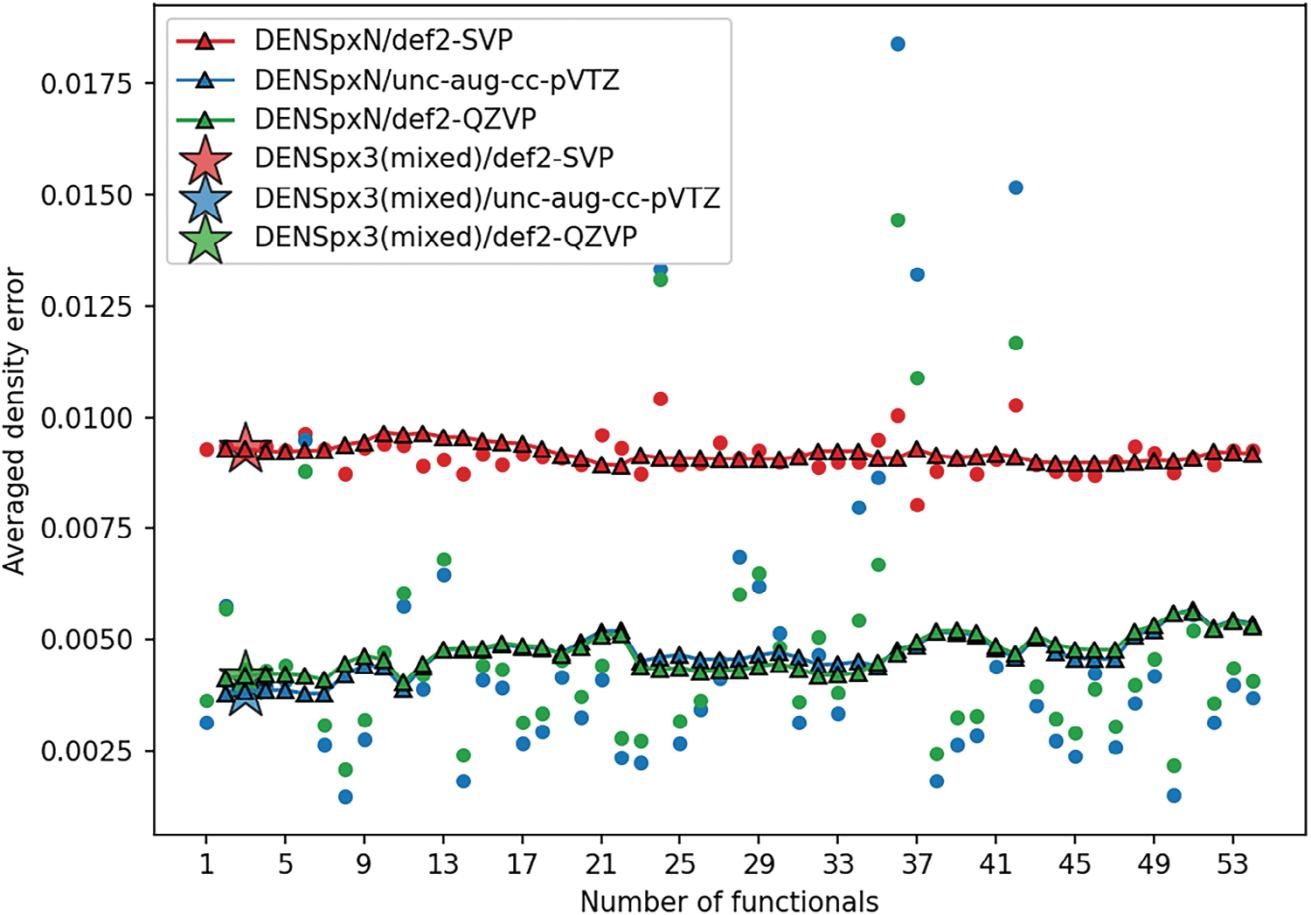
The error of electron densities for each DENS24p×*N* and their component functionals. The color of the marker differentiates the basis set used by DENS24p×*N*: red for def2‐SVP, blue for uncontracted aug‐cc‐pVTZ, and green for def2‐QZVP. The round marker indicates the error of the last functional chosen by DENS24p×*N*. The star marker shows the mixed DENSp×3 functional performance.

Compared with component functionals, the errors of DENS24p×N remain at the median level (sometimes even better, **Figure** **5**). Overall, the ensembles have remarkably stable predictions of electron densities compared to individual functionals: DENS24p×N appears to be immune to outliers, i.e., adding the functional which as large error does not significantly deteriorate the density of the resulting ensemble. The trends in errors of electron densities are rather independent of the basis set. This analysis provides a physical rationale for the robustness of the DENS approach and also gives an idea about how other related properties such as dipoles, partial charges, infrared spectra intensities, etc., can be affected by making ensembles.

**Figure 6:**
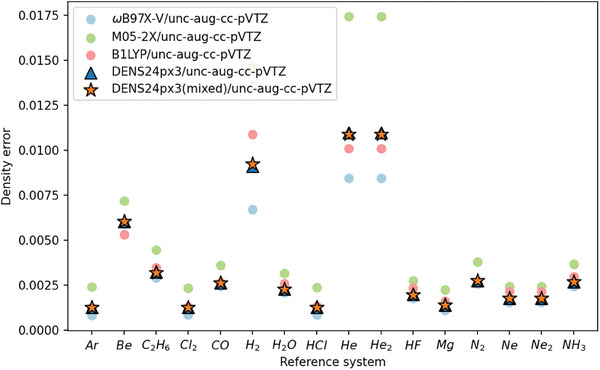
Electron density error of DENS24p×3/unc‐aug‐cc‐pVTZ, its form in mixed functional and component functionals on each reference system.

We dived into each system and checked the performance of DENS24p×3/unc‐aug‐cc‐pVTZ and its component functionals (**Figure** **6**). The highest error appears at H_2_ and He where each component functional tends to exhibit larger errors, e.g. for M05–2X as high as 0.02 on H_2_. We checked the energy profile of DENS24p×3 on reactions containing He in GMTKN55. Most reactions lie in SIE4×4, the datasets for self‐interaction error, which exhibits 11 kcal mol^−1^ error for DENSp×3, suggesting the correlation between the accuracy of DENS for energies and electron densities.

### 4.2 Mixed Density Functionals with Weights from DENS24

Constructing a single‐density functional with tunable parameters for different fractions of exchange‐correlation part is first introduced as a semiempirical strategy for reproducing experimental values in gas‐phase reaction, i.e., the specific reaction parameters (SRP).^[^ ^54^ ^]^ The difference between mixed density functional and our DENS24 is whether the energy fitting process is inside SCF or outside, leading to the nonlinear fitting process and linear one. The reported mixed density functionals were limited to two functionals^[^ ^8^ ^]^ and their weights were reaction specific; the choice of functionals was such that one was overestimating and another underestimating properties for the given system.

The question is whether one can obtain the generalizable mixed functional with the weights for the component functionals obtained through our DENS approach. We explore it by constructing the mixed exchange‐correlation functional as:

(10) $$E_{\mathrm{xc}}^{\mathrm{DENS}}=\sum_{i=1}^{N}{\hat{\omega }}_{i}E_{\mathrm{xc}}^{i}$$

where *i* indicates the *i* ^th^ functional and ${\hat{\omega }}_{i}$ are the normalized weights. We used this functional inside the SCF procedure in contrast to the corresponding DENS functionals were the SCF calculations are performed independently with each functional. As an example, we constructed DENSp×3 (mixed) variant of the DENS24p×3 functional and tested its performance with the def2‐TZVP basis set on the GMTKN55 (we took only 38 subsets due to high computational cost of the evaluations).

**Figure 7:**
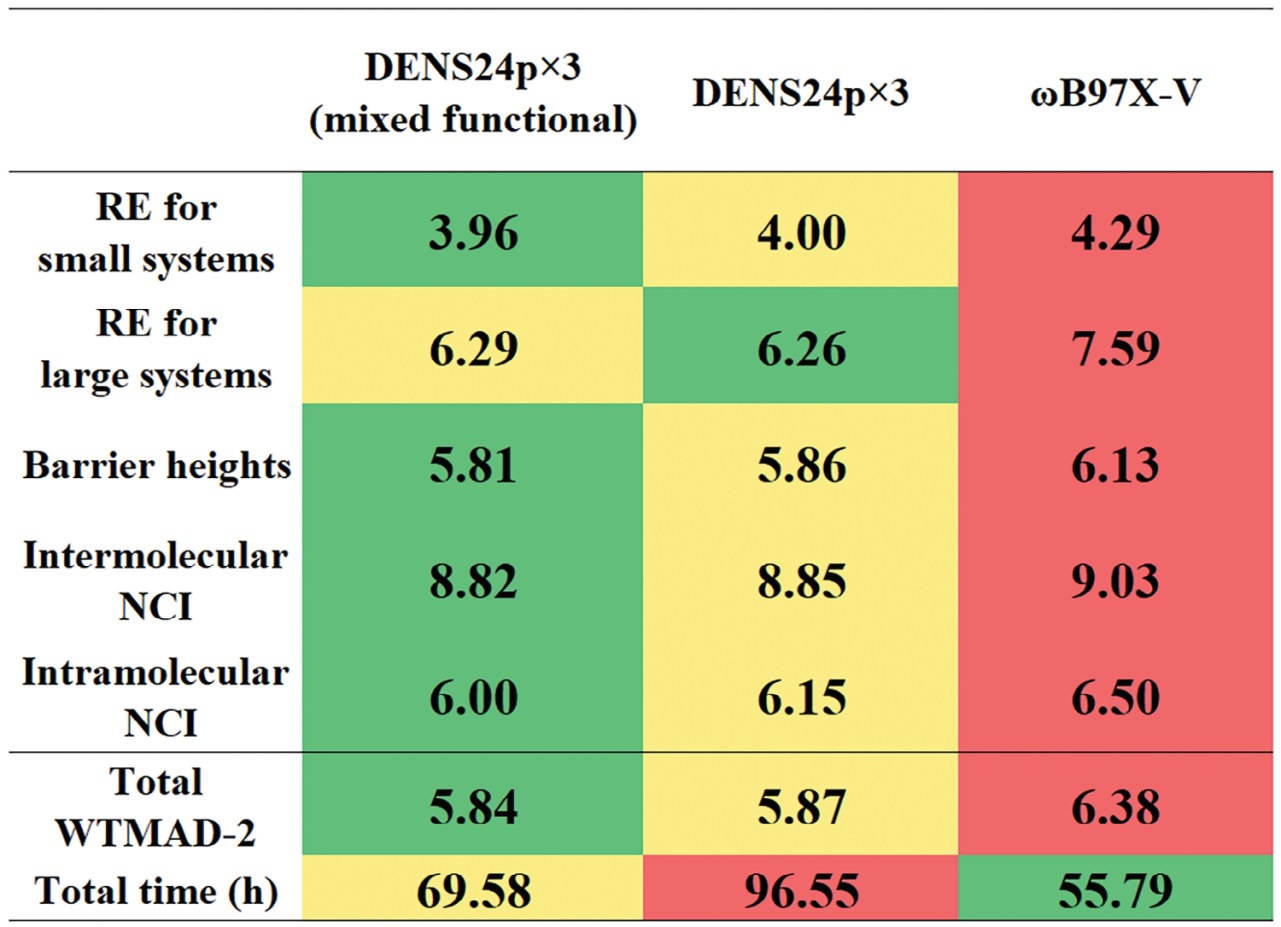
Performance of the DENS24p×3 (mixed) functional WTMAD‐2 in kcal/mol compared with DENS24p×3 and the best hybrid functional ωB97X‐V on 38 subsets of GMTKN55. RE—reaction energies, NCI—noncovalent interactions.

Encouragingly, the mixed functionals have similar (even slightly better) performance than the standard DENS functionals but with a greatly reduced cost as we need to perform only a single SCF calculation with the mixed functional (**Figure** **7**, see Note S5 (Supporting Information) for full results). However, constructing mixed functionals is not as straightforward as building ensembles of functionals and, often, requires careful implementation. However, it can be done at least for three functionals, as we have shown above.

### 4.3 Computational Time for DENS24

**Figure 8:**
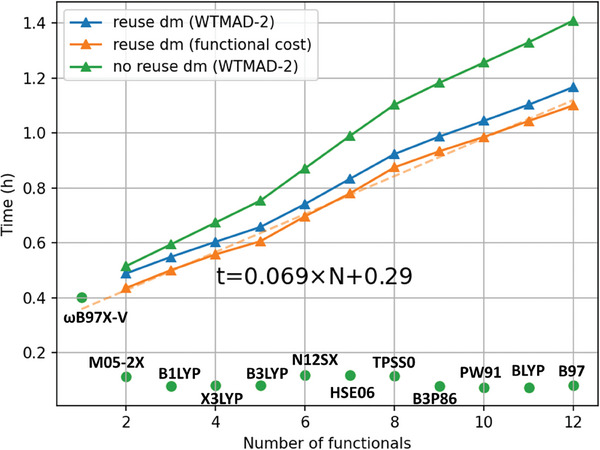
Acceleration of DENS24p×*N* by reusing density matrix from previous functional. The round marker indicates the time cost for the last functional chosen in forward selection. Calculations are performed using 32 CPUs on C_25_H_52_ with def2‐TZVP basis set.

The elephant in the room is the computational cost of the DENS24 functionals. If we perform the SCF calculations completely independently with each functional, the computational CPU time is a sum of the CPU times required for each functional. Luckily, it does not mean that the cost is *N* times larger than, e.g., the best functional. The cost of DFT functionals strongly depends on many factors such as their type (GGA, meta‐GGA, hybrid GGA, *etc*.) and implementation details. Hence, the calculations with DENS24p×12 are only 3.5 times (and not 12 times) larger than that of the first functional in the ensemble for calculating the energy of C_25_H_52_ (**Figure** **8**).

We also suggest two strategies to reduce the cost of DENS24 calculations by reusing the density matrix obtained with one of the functionals as an initial guess for the SCF calculations with the following functional. One strategy is calculating density matrix in the order of accuracy, i.e., the next functional is chosen based on the largest decrease in WTMAD‐2 of GMTKN55. Another is calculating in the order of cost, i.e., the next functional is chosen based on the least cost in the remaining functionals to be used. Both strategies reduce the cost substantially (Figure 8).

As we saw above, creating mixed functionals allows us to perform a single SCF calculation instead of *N* calculations, which can, e.g., costs only 1.25 times (not 3 times) larger than the cost of a single functional for our example with DENS24p×3 (mixed) compared to DENS24p×3 (Figure 7).

In addition, calculations with DENS functionals can be parallelized by performing calculations with each functional on a different computing node, i.e., there is potential to further reduce the wall‐clock time for calculations with the DENS approach.

## 5 Conclusions and Outlook

Here we presented an approach to constructing a more robust DFT‐based methods using an ensemble of DFT functionals instead of focusing on improving a single functional. Such density functional ensembles are by construction more accurate than individual functionals for the properties and systems they are fitted on while retaining good transferability to other systems. The good performance of the ensembles is also evident from a more detailed analysis of all subsets of the GMTKN55 data set, where they are clearly better than constituent functionals (Figure 3).

This study was limited only to the GMTKN55 database and the functionals originally benchmarked on it. Further improvements can be obtained by including more recent, better functionals and their fitting can be done on more diverse data or the data of interest. For example, we might include the excellent DH23 functional in the ensembles in the future and then the error of such functionals will be by construction below 1.76 kcal mol^−1^ (WTMAD‐2 of this single functional).

Anticipating criticism of this work, we can highlight several concerns with the ensemble approach. The obvious drawback is the increased computational cost, but this is the price to pay according to the no free lunch theorem. However, this price can be substantially reduced with the approaches optimizing the computational performance of the ensembles as discussed above. Particularly interesting is creating the mixed exchange‐correlation functionals which allows us to perform single SCF calculation instead of *N* independent calculations. The reader might also wonder why not use machine learning to improve a single functional as it would be faster. Indeed, it is possible but currently, the machine learning‐improved functionals also have limitations and it might be beneficial to construct ensembles of such functionals too.

Concerning DFT, it is conceivable that even better results can be obtained if optimizing the functionals specifically to obtain better results with ensembles of them. Different parametrizations of DFT are usually thrown away and only the best‐performing functionals are reported. Our results indicate that what was considered waste might be very useful in the design of the density functional ensembles. Finally, our approach to constructing ensembles can easily be applied to other methods beyond DFT.

## Conflict of Interest

The authors declare no conflict of interest.

## Author Contributions

Y.R. and Y.C. contributed equally to this work. P.O.D. conceived and designed the project, and participated in methods development and implementation. Y.C. contributed to the initial ideation and tests, and made implementations and benchmarks. Y.R. performed implementations and required calculations. Y.R. and Y.C. performed the major bulk of the analysis of the raw data under supervision by P.O.D. Y.R. and P.O.D. wrote the original version of the manuscript. E.I. contributed to the discussion on the statistical aspects of ensemble learning which shaped the choice of the approach and method development. I.G. contributed to the analysis of the performance of DFT functionals and their ensembles. V.K. and S.S. contributed to the analysis of the electron density. All authors contributed to the discussion of the results and manuscript revisions.

## Supporting information



Supporting Information

Supporting Information

## Data Availability

Data sharing is not applicable to this article as no new data were created or analyzed in this study. The code with the Python implementation of the DENS24p×*N* and DENS24o×*N* ensembles is available in the open‐source MLatom at https://github.com/dralgroup/mlatom.
