# Supplementary material for: The Best DFT Functional Is the Ensemble of Functionals
Source: Adv Sci (Weinh). 2024 Oct 25;11(47):2408239. doi: 10.1002/advs.202408239 (PMC11653610; doi:10.1002/advs.202408239)
Supplement: Supplementary file 1 — Supporting Information [file ADVS-11-2408239-s002.pdf]

ADVANCED SCIENCE
Open Access

Supporting Information

for *Adv. Sci.*, DOI 10.1002/advs.202408239

The Best DFT Functional Is the Ensemble of Functionals

*Yuting Rui, Yuxinxin Chen, Elena Ivanova, Vignesh Balaji Kumar, Szymon Śmiga, Ireneusz Grabowski and Pavlo O. Dral**

Supplementary Information for "The best DFT functional is the ensemble of functionals"

Yuting Rui,[1] Yuxinxin Chen,[1] Elena Ivanova,[2] Ireneusz Grabowski,[3] Pavlo O. Dral[1,3*]

[1]*State Key Laboratory of Physical Chemistry of Solid Surfaces, College of Chemistry and Chemical Engineering, Fujian Provincial Key Laboratory of Theoretical and Computational Chemistry, Xiamen University, Xiamen, Fujian 361005, China*

[2]*Chair of Statistics, School of Business and Economics, Humboldt University of Berlin, Unter den Linden 6, 10099 Berlin, Germany*

[3]*Institute of Physics, Faculty of Physics, Astronomy, and Informatics, Nicolaus Copernicus University in Toruń, ul. Grudziądzka 5, 87-100 Toruń, Poland*

Email: dral@xmu.edu.cn

SI note 1. Boxplot of error of DENS24×*N* and representative functionals on GMTKN55

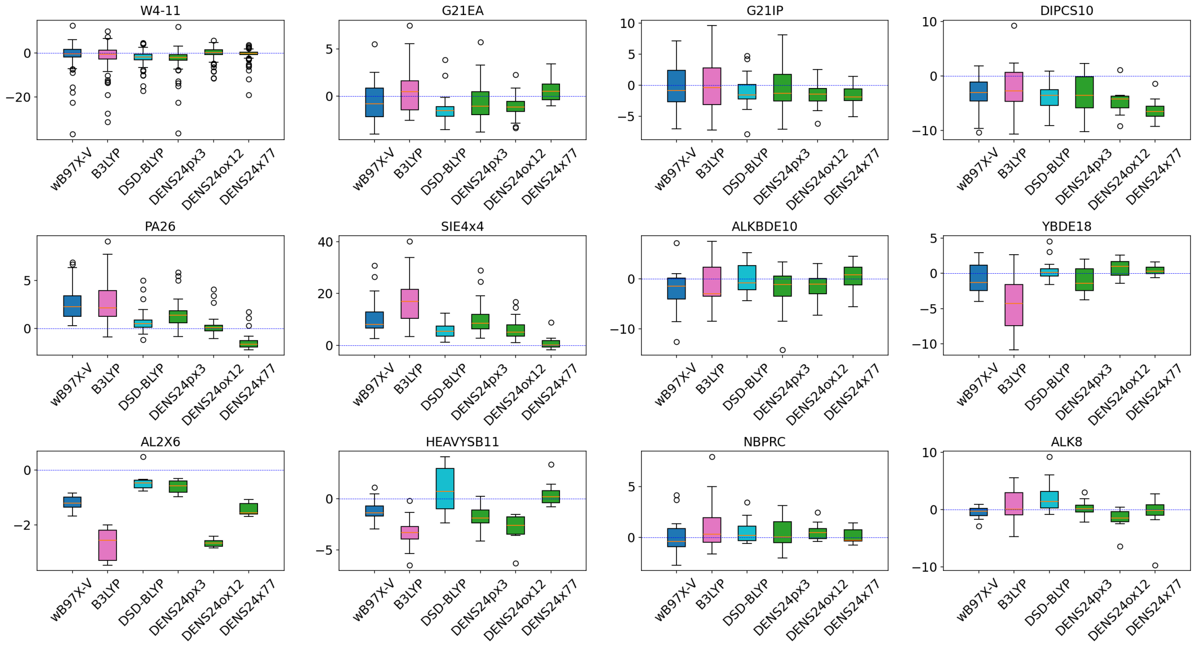

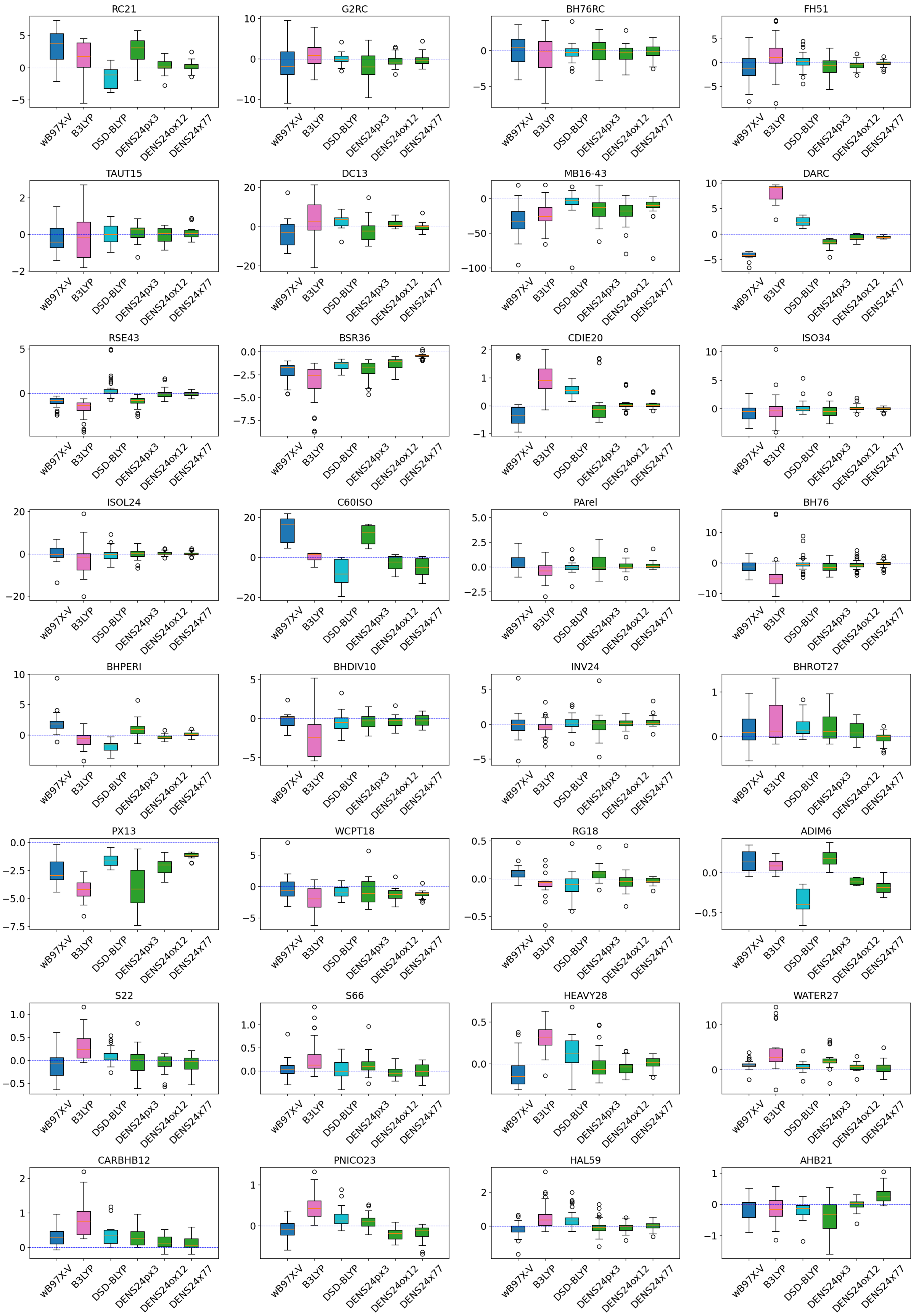
RC21
G2RC
BH76RC
FH51
TAUT15
DC13
MB16-43
DARC
RSE43
BSR36
CDIE20
ISO34
ISOL24
C60ISO
PArel
BH76
BHPERI
BHDIV10
INV24
BHROT27
PX13
WCPT18
RG18
ADIM6
S22
S66
HEAVY28
WATER27
CARBHB12
PNICO23
HAL59
AHB21
wB97X-V
B3LYP
DSD-BLYP
DENS24px3
DENS24ox12
DENS24x77

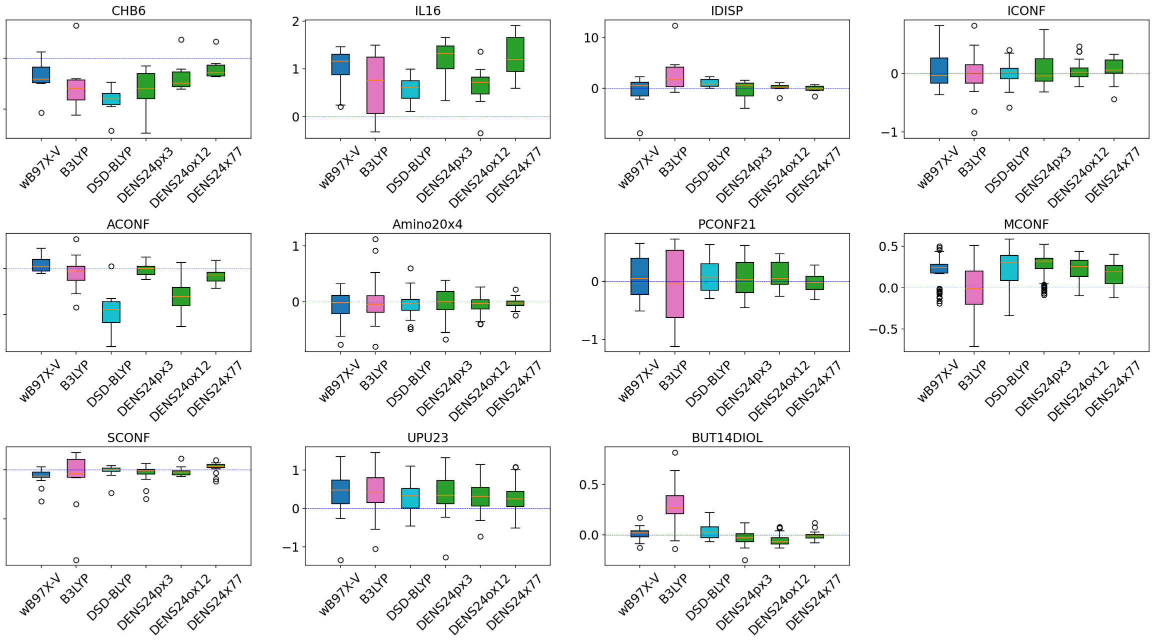

SI note 2. Boxplot of error of DENS24p×42 (the rightmost) and component functionals on GMTKN55

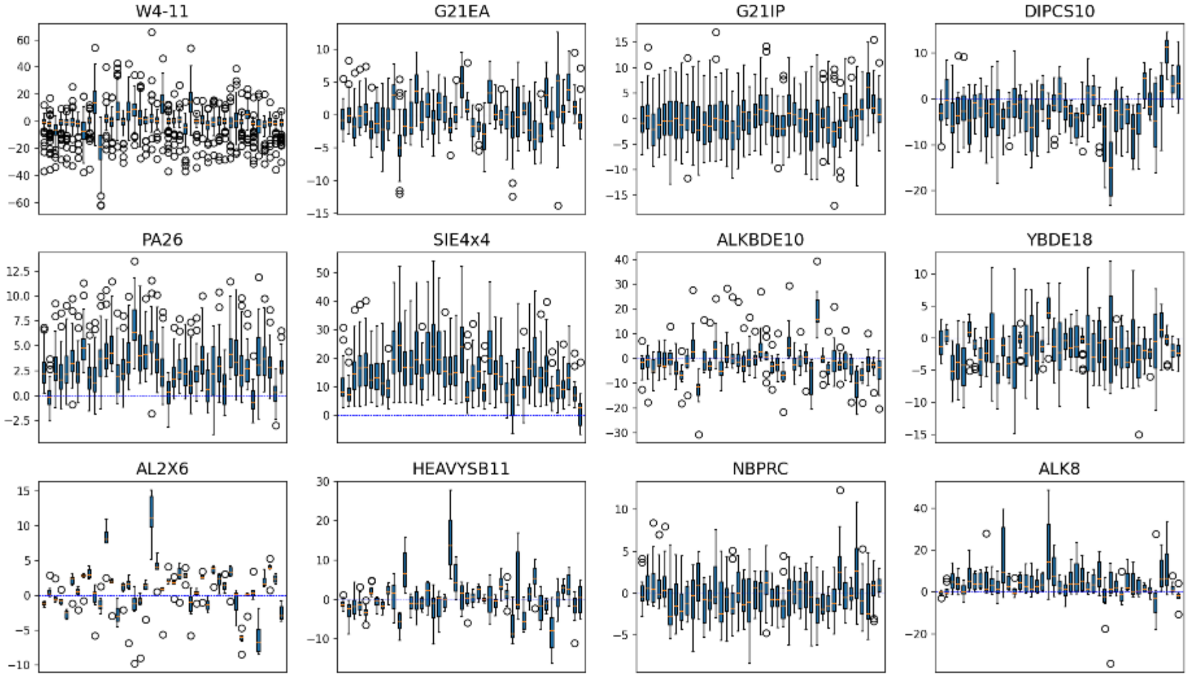

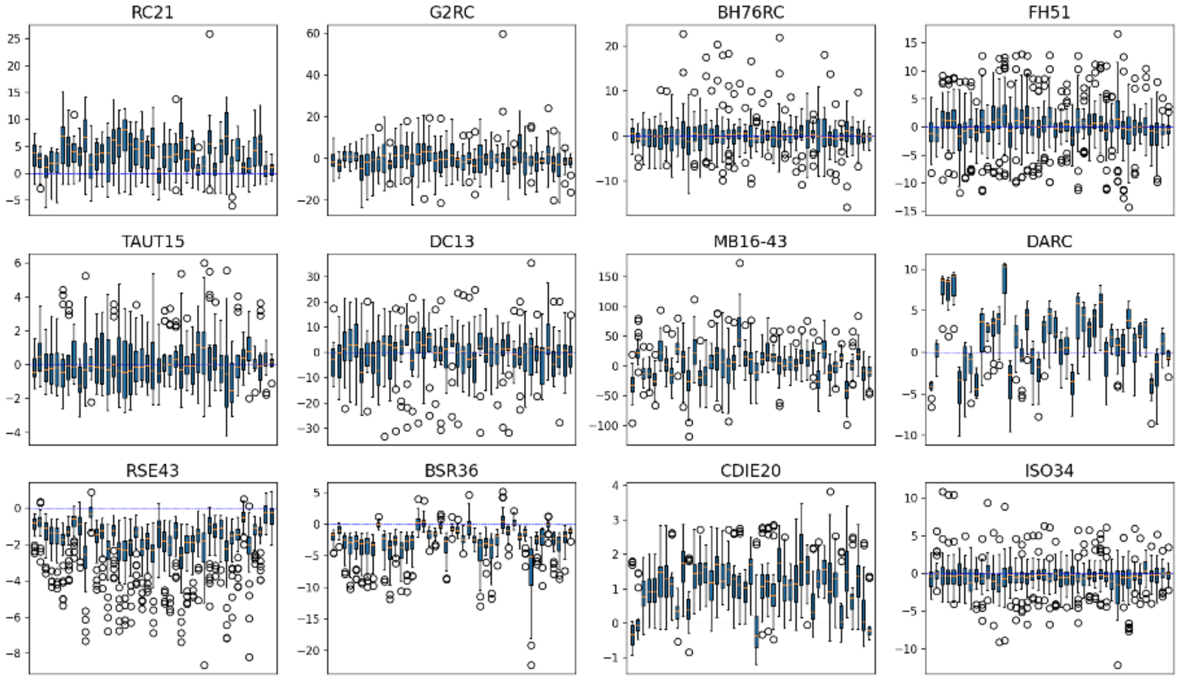
RC21
G2RC
BH76RC
FH51
TAUT15
DC13
MB16-43
DARC
RSE43
BSR36
CDIE20
ISO34

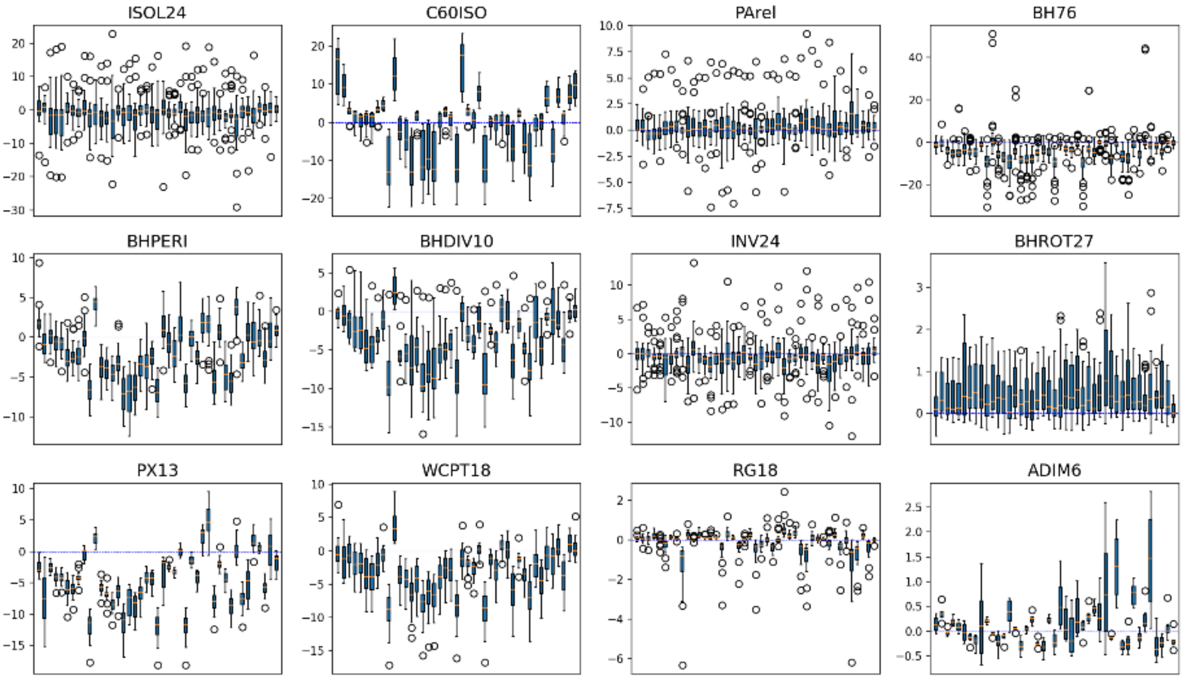
ISOL24
C60ISO
PArel
BH76
BHPERI
BHDIV10
INV24
BHROT27
PX13
WCPT18
RG18
ADIM6

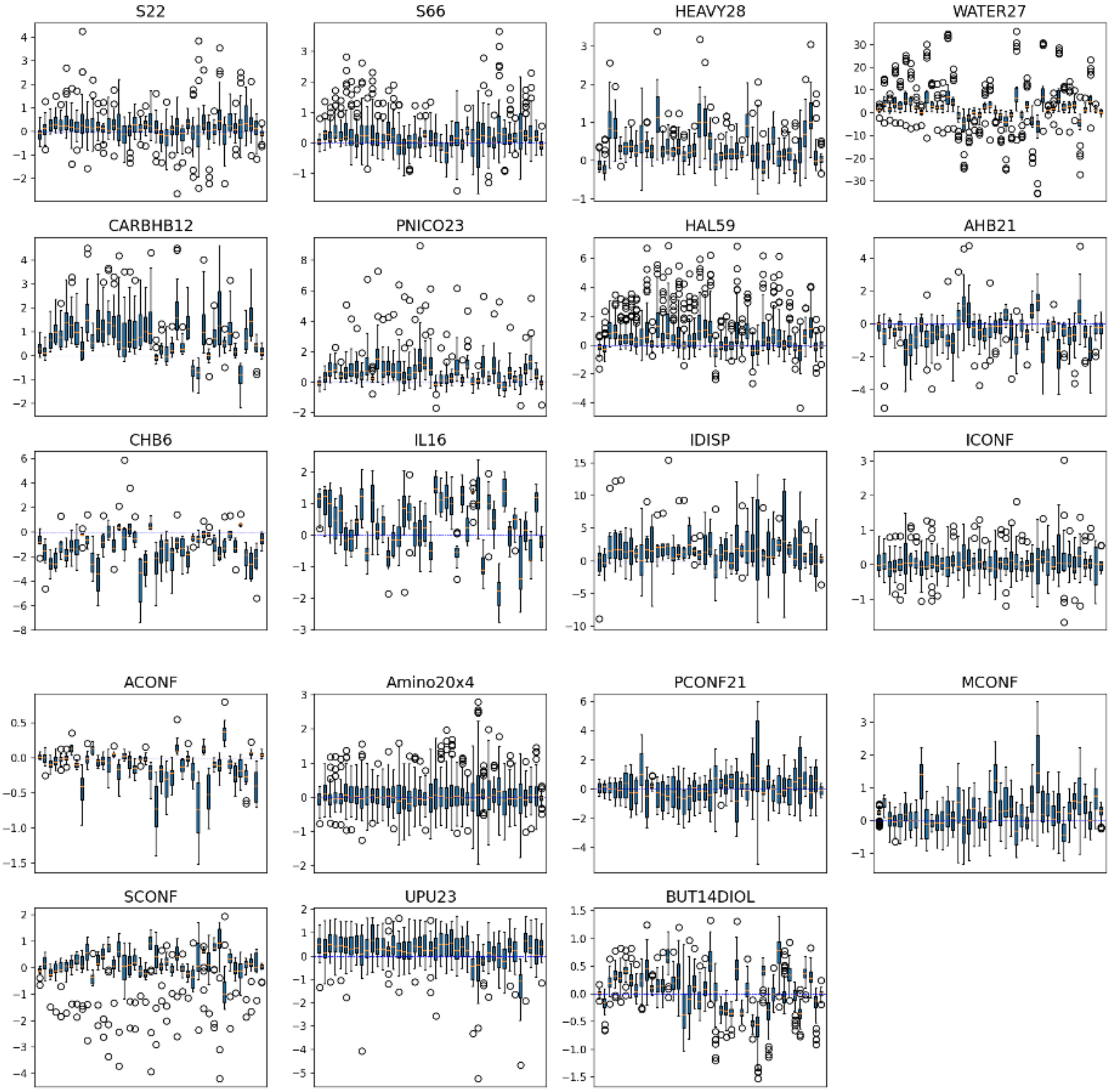

SI note 3. Comparison of DENS24p×3, DENS24p×12 and DENS24p×42 on GMTKN55

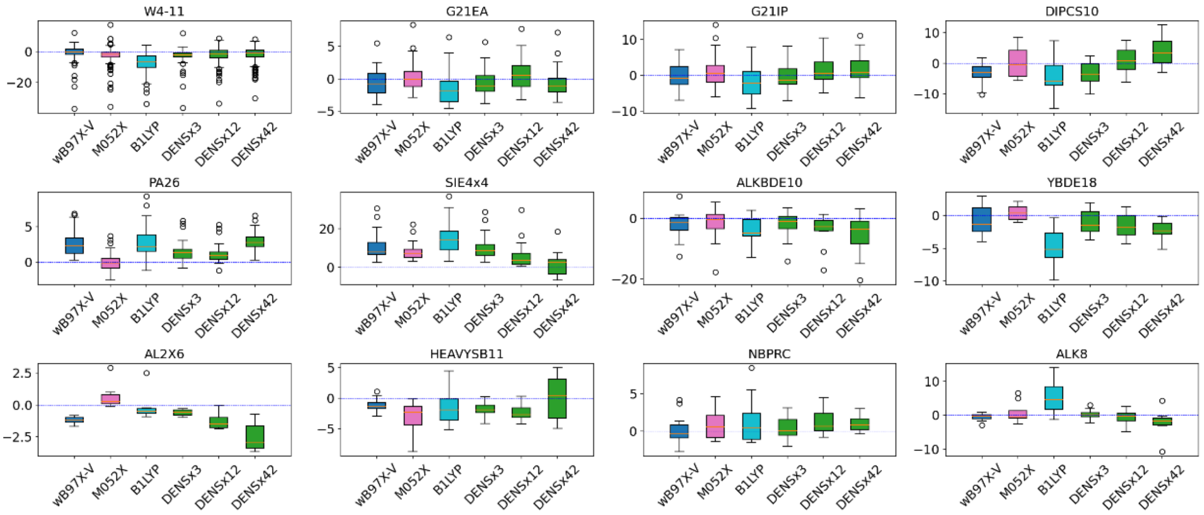

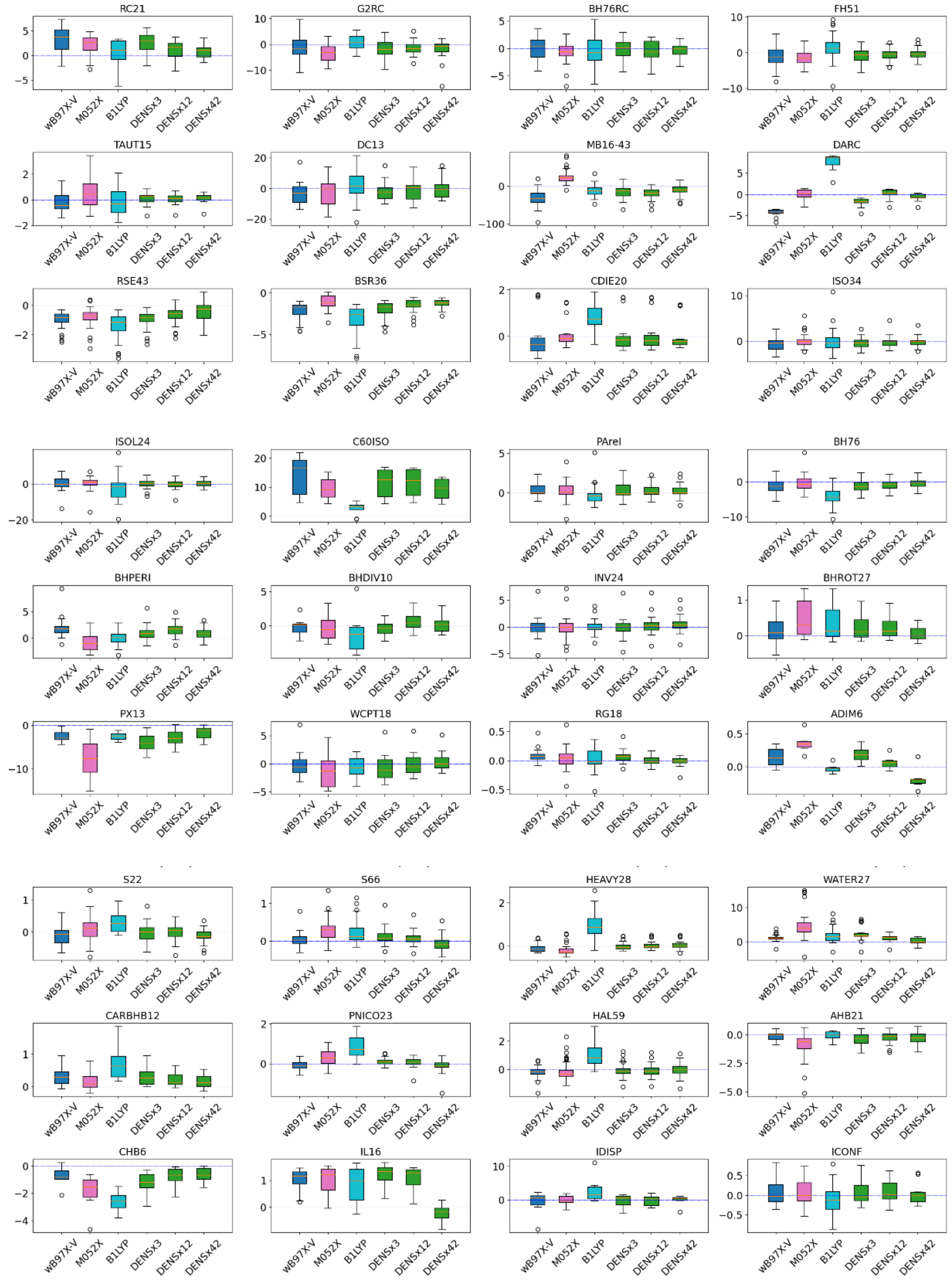
RC21
G2RC
BH76RC
FH51
TAUT15
DC13
MB16-43
DARC
RSE43
BSR36
CDIE20
ISO34
ISOL24
C60ISO
PArel
BH76
BHPERI
BHDIV10
INV24
BHROT27
PX13
WCPT18
RG18
ADIM6
S22
S66
HEAVY28
WATER27
CARBHB12
PNICO23
HAL59
AHB21
CHB6
IL16
IDISP
ICONF
wB97X-V
M052X
B1LYP
DENSx3
DENSx12
DENSx42

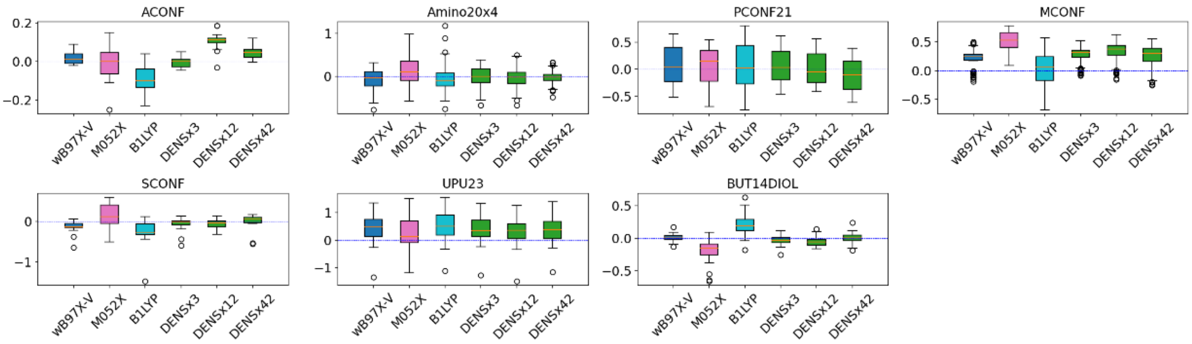

SI note 4. Dissociation curve of methane

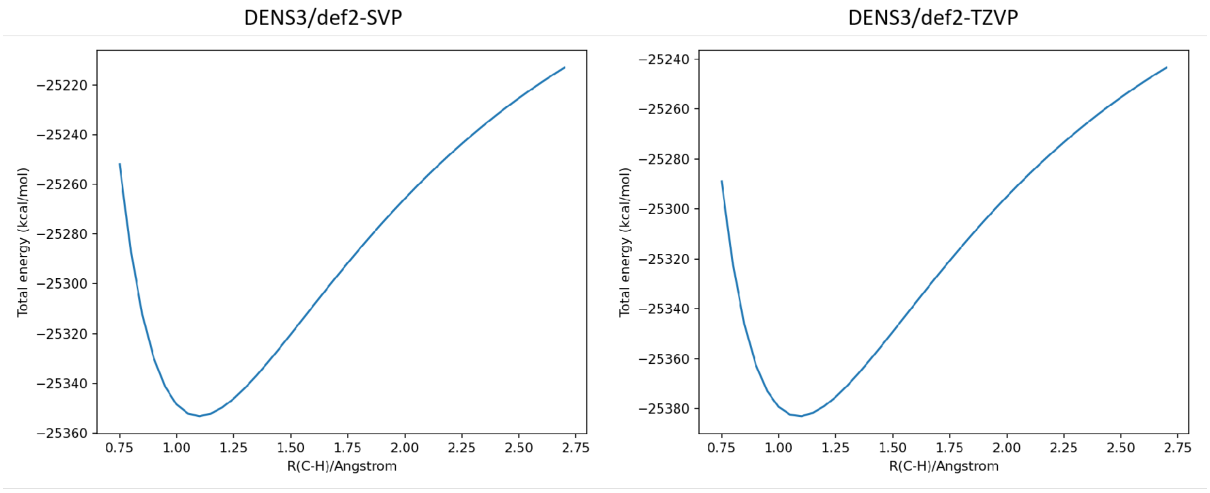

SI note 5. Performance of DENS24p×3 (with and without mixed functional scheme) and its component functionals on 38 subsets in GMTKN55 with def2-TZVP basis set.

subset	DENS24p×3/ def2-TZVP (mixed functional)	DENS24p×3/ def2-TZVP	ωB97X-V/ def2-TZVP	M05-2X-D3/ def2-TZVP	B1LYP-D3/ def2-TZVP
basic properties and reaction energies for small systems					
W4-11	3.36	3.76	3.12	4.32	8.69
G21EA	7.57	7.56	7.88	6.31	9.70
DIPCS10	4.06	4.46	4.21	3.88	6.04
SIE4x4	11.12	11.06	11.47	8.71	16.03
ALKBDE10	5.57	5.64	5.33	5.68	7.30
YBDE18	1.53	1.50	1.81	1.03	5.28
AL2X6	0.82	0.86	1.11	0.74	3.32
NBPRC	0.92	0.93	1.31	1.32	1.96
ALK8	1.94	1.91	1.42	2.51	6.09
G2RC	3.59	3.57	4.56	3.38	4.00
BH76RC	2.08	2.08	2.49	1.61	2.78
WTMAD-2	3.96	4.00	4.29	3.48	6.15
Time (h)	5.37	6.29	3.67	1.98	0.64
reaction energies for large systems and isomerisation reactions					
MB16-43	20.79	20.98	35.68	21.71	36.07
DARC	2.39	2.36	4.81	1.02	7.11
RSE43	1.00	0.99	1.03	0.81	1.44
BSR36	2.06	2.05	2.20	1.33	3.02
CDIE20	0.47	0.47	0.62	0.33	1.04
ISO34	1.18	1.18	1.38	1.15	2.02
PArel	0.68	0.68	0.75	1.05	0.88
WTMAD-2	6.29	6.26	7.59	5.57	10.37
Time (h)	23.87	33.25	18.68	9.72	4.85
reaction barrier heights					
BH76	2.19	2.23	2.32	2.06	4.91
BHPERI	1.22	1.18	1.97	1.54	0.81
BHDIV10	0.90	0.87	0.92	1.43	2.38
BHROT27	0.38	0.38	0.37	0.52	0.46
PX13	5.25	5.39	3.96	8.79	4.08
WCPT18	2.60	2.63	2.50	3.04	2.41
WTMAD-2	5.81	5.86	6.13	6.62	9.75
Time (h)	8.14	10.74	6.23	3.24	1.26
intermolecular noncovalent interactions					
RG18	0.25	0.25	0.28	0.26	0.10
ADIM6	0.20	0.21	0.15	0.56	0.30
S22	0.35	0.35	0.32	0.49	0.70
WATER27	14.46	14.45	14.07	15.30	15.84
CARBHB12	0.54	0.55	0.57	0.44	0.90
PNICO23	0.42	0.42	0.38	0.65	0.63
AHB21	3.58	3.56	3.48	3.79	3.79
CHB6	1.57	1.55	1.42	2.09	1.96
IL16	2.24	2.10	2.43	1.73	3.42
WTMAD-2	8.82	8.85	9.03	10.03	8.60
Time (h)	13.27	17.87	10.83	4.55	2.49
intramolecular noncovalent interactions					
IDISP	1.63	1.60	2.55	1.27	2.98
ICONF	0.24	0.25	0.22	0.32	0.32
ACONF	0.04	0.05	0.03	0.09	0.07
SCONF	0.38	0.39	0.34	0.43	0.60
BUT14DIOL	0.34	0.35	0.38	0.21	0.80
WTMAD-2	6.00	6.15	6.50	4.94	12.61
Time (h)	18.93	28.40	16.37	7.63	4.40
GMTKN55					
Total WTMAD-2	5.84	5.87	6.38	5.73	8.93
Total time (h)	69.58	96.55	55.79	27.12	13.64
